# Decision to take osteoporosis medication in patients who have had a fracture and are 'high' risk for future fracture: A qualitative study

**DOI:** 10.1186/1471-2474-12-92

**Published:** 2011-05-09

**Authors:** Joanna EM Sale, Monique A Gignac, Gillian Hawker, Lucy Frankel, Dorcas Beaton, Earl Bogoch, Victoria Elliot-Gibson

**Affiliations:** 1 Mobility Program Clinical Research Unit, Li Ka Shing Knowledge Institute, St. Michael's, Toronto, Ontario, Canada; 2 Department of Health Policy, Management & Evaluation, University of Toronto, Toronto, Ontario, Canada; 3 Toronto Western Research Institute, University Health Network, Toronto, Ontario, Canada; 4 Dalla Lana School of Public Health, University of Toronto, Toronto, Ontario, Canada; 5 Department of Medicine, University of Toronto, Toronto, Ontario, Canada; 6 Osteoporosis Research Program, Women's College Hospital, Toronto, Ontario, Canada; 7 Mobility Program, St. Michael's, Toronto, Ontario, Canada; 8 Department of Surgery, University of Toronto, Toronto, Ontario, Canada

## Abstract

**Background:**

Patients' values and preferences are fundamental tenets of evidence-based practice, yet current osteoporosis (OP) clinical guidelines pay little attention to these issues in therapeutic decision making. This may be in part due to the fact that few studies have examined the factors that influence the initial decision to take OP medication. The purpose of our study was to examine patients' experiences with the decision to take OP medication after they sustained a fracture.

**Methods:**

A phenomenological qualitative study was conducted with outpatients identified in a university teaching hospital fracture clinic OP program. Individuals aged 65+ who had sustained a fragility fracture within 5 years, were 'high risk' for future fracture, and were prescribed OP medication were eligible. Analysis of interview data was guided by Giorgi's methodology.

**Results:**

21 patients (6 males, 15 females) aged 65-88 years participated. All participants had low bone mass; 9 had OP. Fourteen patients were taking a bisphosphonate while 7 patients were taking no OP medications. For 12 participants, the decision to take OP medication occurred at the time of prescription and involved minimal contemplation (10/12 were on medication). These patients made their decision because they liked/trusted their health care provider. However, 4/10 participants in this group indicated their OP medication-taking status might change. For the remaining 9 patients, the decision was more difficult (4/9 were on medication). These patients were unconvinced by their health care provider, engaged in risk-benefit analyses using other information sources, and were concerned about side effects; 7/9 patients indicated that their OP medication-taking status might change at a later date.

**Conclusions:**

Almost half of our older patients who had sustained a fracture found the decision to take OP medication a difficult one. In general, the decision was not considered permanent. Health care providers should be aware of their potential role in patients' decisions and monitor patients' decisions over time.

## Background

Individuals who are 65+ years and have sustained a fragility fracture are at increased risk for future fracture [1-4] and should be investigated for underlying osteoporosis (OP) [1,2]. If bone densitometry confirms reduced bone mass, and thus high risk for fracture if untreated, antiresorptive medication should be considered to reduce this fracture risk [1,2]. However, once OP medication is prescribed, its effectiveness depends primarily on patient adherence. There is an abundance of literature to suggest that in real world settings, treatment adherence in OP patients after a fracture is low [5-9]. For example, up to 58% of patients with a fracture who have an indication for treatment (low bone density on bone densitometry) and have been recommended OP medication by a physician are not taking an antiresorptive medication at 6 months following the recommendation [8]. Further, approximately 74% of patients who have sustained a fracture and have been screened through a post-fracture OP intervention discontinue the medication within 12 months of initiating it [10].

Patients' values and preferences are fundamental tenets of evidence-based practice, yet current OP clinical practice guidelines pay little attention to these issues in therapeutic decision making [11]. This may be in part due to the fact that few studies have examined the factors that influence the initial decision to take OP medication. One study investigated *preferences *in older women by presenting them with a description of different OP medications and found that side effects and the cost of medication were factors considered by women when they discussed treatment selection and medication use [12]. However, the study was conducted in a community sample of women who were not on treatment for OP but were rather taking medication for other conditions (e.g. arthritis, cardiovascular disease). Conversely, Weiss and colleagues [13] found that effectiveness was ranked as the most important determinant of *preference *in women with, or at risk for, OP when they were presented with attributes of various OP medications (e.g. effectiveness, dosing regimen, how long the drug had been on the market). The purpose of our study was to investigate patients' experiences with the *actual decision *to take prescribed OP medication after being screened post-fracture for OP in a university teaching hospital.

## Methods

### Study Design

The experience of decision-making lends itself to a phenomenological study [14,15]. Phenomenology is a qualitative approach where individuals are asked to focus on a specific situation that they actually experienced and to describe it in sufficient detail so that new knowledge about the phenomenon can be obtained [16]. Specifically, an eidetic phenomenological study was conducted which assumes there are structures, or commonalities, to patients' experiences regarding their decision to take OP medication [15]. The purpose of this type of inquiry is to describe this common structure based on a diversity of experiences [14].

### Recruitment

Using phenomenological methods, the meaning of the decision to take medication was examined using accounts of individuals who had "lived through" this experience [17]. Sampling was purposeful and included male and female patients who had sustained a fracture, were prescribed OP medication, and were able to articulate their experiences (e.g. did not demonstrate cognitive impairment). Participants were identified from among patients screened by a coordinator through a fracture clinic OP screening program [18] at an urban teaching hospital. Eligibility criteria were English-speaking outpatients aged 65+ with, or without, a history of OP treatment who had sustained a fragility fracture in the last 5 years, were deemed "high risk" for future fracture based on age, sex, prior fracture, and BMD values [19], and were prescribed OP medication. The Ontario Drug Benefit plan covers OP medication for individuals aged 65+ so cost of treatment was not a barrier to accepting treatment for our sample.

Patients who met our eligibility criteria were asked by the coordinator if they were interested in participating in a study on post-fracture care. Study staff contacted those who agreed. Study approval was obtained from the hospital's Research Ethics Board. All patients gave their informed consent prior to their inclusion in the study.

### Data Collection

Data were collected through face-to-face semi-structured interviews in patients' homes. If a home interview was not convenient, the patient was interviewed in the researcher's office, or by telephone. Interviews were 1-2 hours in duration and were audiotaped. Referring to an interview guide (see Table 1), the interviewer asked the patient to talk about their fracture and their experience in the fracture clinic. Patients were then asked about recommendations for their bone health received by specialists/general practitioners (GPs) as well as their responses to recommendations.

**Table 1:** Interview Guide

Interview Protocol
1. Tell me about your fracture.
2. What recommendations did you receive by your health care provider(s) regarding your bone health after your fracture? Probe: What was your reaction to those recommendations? Probe: What about the medication that was prescribed to you?
3. What are you doing about those recommendations?
4. What has your GP/specialist explained to you about your bone health? Probe: What is your relationship with your GP/specialist?
5. What motivates you to take/not take your OP medication? Probe: What makes it difficult?
6. What (or who) made you decide to take/not take your medication? Explain.

Consistent with the concept of "bracketing" in eidetic phenomenology (the setting aside of one's judgments, biases, and preconceived ideas about the phenomenon) [20-23], the interviewer remained as neutral as possible throughout the interview and did not reinforce or discourage any topics discussed related to OP medication use. For example, patients often discussed recommendations and experiences regarding general bone health management, not only medication use.

All interviews were taped with a digital recorder and detailed notes were taken. Interviews were transcribed and downloaded in NVivo 7 [24], a qualitative software program with flexible features that helped organize, code, and retrieve data. Patient's age, sex, bone densitometry T-scores (at the total hip, femoral neck, and L1-L4), fracture type, fracture history, time since most recent fracture, and OP medication status were recorded. While we report each patient's age and sex and bisphosphonate use, we have only described fracture type, T-score, and time since fracture for the group as a whole in order to protect participants' identities.

### Data Analysis

Two researchers (JS, LF) coded all transcripts to promote a comprehensive examination of the data. These researchers met after each interview to discuss the content of the interview and to revise the interview guide in order to capture additional topics of interest. Data analysis was guided by Giorgi's methodology [25,26]. Analysis began after the first interview and was an iterative process with "meaning units" (codes) identified immediately and then revised as more interviews were conducted. A coding template was developed; after six revisions, the two researchers agreed on a final template. Interrelationships among codes were examined and a common structure of the decision to take OP medication was synthesized. Direct quotations from the transcripts illustrated our findings.

Consistent with the phenomenological concept of "imaginative variation" [20,21], multiple thematic possibilities were considered during analysis. Discussion of these themes occurred at the level of the larger research team. The funding source had no role in this study.

## Results

Thirty patients were identified and 21 (6 males, 15 females) consented to participate in our study. This sample size meets recommendations for phenomenological studies [14,27]. Reasons for not participating included too ill (n = 2), not interested (n = 6), and caring for a sick spouse (n = 1). Consistent with qualitative research, five patients were interviewed a second time by telephone to clarify information in their transcripts, thus yielding 26 interviews. Seventeen of the original 21 interviews were conducted in patients' homes, 3 were conducted in the researcher's office, and 1 was conducted by telephone. Table 2 describes the study sample as a whole. Participants ranged in age from 65 to 88 and fracture type included 7 wrist, 7 hip, 4 shoulder, 1 ankle, 1 patella, and 1 pelvic fracture. Time since the most recent fracture ranged from 6-12 months (n = 12), 12-18 months (n = 7), to 2-5 years (n = 2). All participants had low bone mass (DXA T-score < -1.0); 9 had OP (T-score ≤ -2.5) [28,29]. There were no trends noted in our findings between the decision to take OP medication and age, gender or fracture location. At the time of the interview, 14 patients were taking a bisphosphonate; the remaining 7 patients were taking no OP medication.

**Table 2:** Description of Study Sample (n = 21)

Characteristic		
Age, yrs, range		65-88
Female, n (%)		15 (71)
Fracture type, n %)		
	Wrist	7 (33)
	Hip	7 (33)
	Shoulder	4 (19)
	Ankle	1 (5)
	Patella	1 (5)
	Pelvis	1 (5)
Time since most recent fracture, n (%)		
	6-12 months	12 (57)
	12-18 months	7 (33)
	2-5 years	2 (10)
T-Score, n (%)		
	-1.0 to -2.4	12 (57)
	≤ -2.5	9 (43)
Taking bisphosphonate at time of interview, n (%)		14 (67)
Decision to take OP medication, n (%)		
	"Easy"	12 (57)
	"Difficult"	9 (43)

For 12 participants, the decision to take, or not take, OP medication was relatively easy, involved minimal contemplation or distress, and occurred at the time the prescription was given. Ten of these 12 patients were taking OP medication; 1 patient had initiated and then stopped taking OP medication and 1 patient had not initiated OP medication (had not filled his prescription). For 9 patients, the decision to take, or not take, OP medication was more difficult, requiring time and consideration of several factors. Four of these 9 patients were taking OP medication while 5 patients were not taking medication. The following section describes these two decision categories in further detail.

### Decision required minimal contemplation ('easy' decision)

Patients in this group described the decision as an easy one, which required minimal contemplation. For example, one participant considered his bisphosphonate to be a "minor medication...just more like supplements than medication" (#20-male age 65). Patients reported making the decision to take, or not take, medication at the time it was prescribed for them, speaking highly of their health care provider. Participants indicated that they "liked" their health care provider (GP or specialist) (#9-female age 68, #17-male age 79, #20-male age 65), had "a great relationship" with their health care provider (#13-female age 70), or "trusted" their health care provider in prescribing the bisphosphonate (#18-female age 73, #16-female age 75, #11-male age 73). For example, participant #11 told us that he valued his specialist's "expertise and judgment and follow[ed] whatever recommendations he [was] given without question" even though he did not really understand the purpose of the OP medication. Similarly, one 82-year old male patient who quickly decided *not *to take OP medication was influenced by the opinion of his GP who he described as "well informed". He recalled that his GP had warned him of the "complications" of taking OP medication, saying that it was "no great treat...swallowing it, you have to be careful it doesn't lodge in your throat. You have to be standing up or something, I forget" (#19).

In general, participants in the "easy decision" group made their decisions based on trust in their health care provider rather than trust in the OP medication. When asked about the OP medication itself, participants in this group made brief statements about the perceived benefits of the medication as "[keeping bone] from weakening" (#2-male age 88), providing "extra strength for the bone" (#9-female age 68), "[preventing] further bone loss" (#25-female age 87), or "[improving]...bone density" (#11-male age 73). With the exception of one patient who complained about stomach upset, patients taking OP medication at the time of the interview who found the decision to take an easy one did not articulate any potential, or actual, risks of OP medication, suggesting that they did not perform any risk-benefit analyses about the medication.

Although the decision to take OP medication had been relatively easy for all 12 participants in this category, 4 participants indicated that their current medication status might change. Three of the 10 patients who were taking OP medication (#2-male age 88, #6-female age 71, and #17-male age 79) were not convinced about their decision to take the medication, suggesting that they might subsequently decide *not *to take it. For example, one patient did not understand what the medication was for at all and did not appear to take it regularly, "I think from time to time there was some pill or something that I had to take with each meal or with a meal during any one day...we aren't always getting up in the morning thinking about pills" (#2-male age 88). His wife helped him with his medication regimen but she admitted that he missed taking pills and that he often "erred and strayed from [his] ways". Another patient told us she "came from an age where people [did] what they were told". However, a friend had informed her that there was not much one could do to reverse the deterioration of bone: "it doesn't matter what you do. There is not a lot you can do to change what happened" (#6-female age 71).

Another participant had been diagnosed with OP 20 years previously and had his bisphosphonate prescription refilled regularly by his GP. However, he never discussed his bone health with his GP. This participant told us he often forgot to take his bisphosphonate because he "couldn't measure what [it was] doing for [him]". If he forgot to take it, he told us that he didn't worry about or "miss it" (#17-male age 79).

Similarly, one participant *stopped *taking prescribed OP medication after her first prescription ran out due to stressful life events (including a death in the family and a change of residence) (#25-female age 87). While she described herself as from a generation who followed doctors' orders without questioning them, she told us: "when your routine is completely destroyed, when every day...changes, it's difficult to stay on a routine." She indicated she would probably start taking a bisphosphonate again in the future, "Yeah,...I'm going to get back on to it [medication]."

### Decision required considerable contemplation ('difficult' decision)

Participants classified under this group described the decision to take, or not take, OP medication as more difficult. Four of these 9 patients were taking OP medication at the time of the interview (4 females age 70-84) and 5 were *not *taking medication at the time of the interview (1 male age 65, 4 females age 65-77). The role of the health care provider in this group was also influential. However, while some participants trusted/respected their health care providers, they wanted additional discussion and reported needing to be *convinced *by them in order to make a decision (see Table 3). For example, one participant stated, "he [specialist] didn't say anything that convinced me that I needed to take the medication" (#22-female age 65).

**Table 3:** Examples of the perceived role of health care providers in the decision to take OP medication

"Easy" Decision		"Difficult" Decision	
Taking OP medication at time of interview	Not taking OP medication at time of interview	Taking OP medication at time of interview	Not taking OP medication at time of interview

"If she [specialist] had said to me, I want you to drink three gallons of orange juice a day, I probably would have said, okay"... *and later*, "I have considerable respect for the man [GP] that I see" (#11)	"I find my medical doctor, he's good. He seems to be well-informed...he advised me not to take it" (#19)	"it was my second visit and he [specialist] was every so kind. He held my hand and he told me we can't take any more chances with any more fractures...and I thought all of that through" (#21)	"he [specialist] didn't say anything to me that convinced me that I needed to take medication" (#22)

Patients in this group who were taking medication reported that their GP or specialist had been able to influence their decision and convince them to take it. One 71-year old female patient told us that on her second visit with her specialist, he answered all her questions and made everything clear, "These pills would help build my bones and I thought all of that through...I thought, he's a doctor. He knows what he's talking about. If it's going to help me, what am I going to do? Just let them sit on that dresser? That would be silly. Take them and try to get better." (#21)

Another female patient, aged 70, with two prior fractures (#10), described concerns about taking bisphosphonates. Her general practitioner had prescribed OP medication following a previous BMD test but she had been "turned off" by her physician's attitude: "I got the impression from her [the GP] that she automatically put women on bone density medication once they were fifty or over...So I was not convinced to take it because...I wasn't convinced that I needed it. Not at all." She was then referred to a specialist who gave her an in-depth explanation of her condition and about the medication itself. Following the visit with her specialist, this participant decided to take OP medication: "I felt very confident and secure once I spoke with her [the specialist] in detail about my concerns taking the drug. I just didn't want to take any drug unless it was necessary. But she explained everything so thoroughly and had information to back it up from my charts. So she convinced me and she said she doesn't mainly prescribe drugs either, nor does she like taking them herself unless it's necessary."

A third participant (#15-female age 84) had presented with a second fracture to the fracture clinic; she described her specialist as "condescending and not direct enough" and had not been able to understand the OP medication prescription she had received after her first fracture: "I couldn't make head or tail of it and nor could my pharmacist". She had actually left her specialist's office prior to completing her appointment after her first fracture. However, the second fracture heightened her perception of the seriousness of her bone health. At this point, her GP explained the recommended treatment more clearly and threatened that she'd have to stop smoking if she didn't take her OP medication: "he [the GP] said you either take that or I get you right off your smoking immediately (laughter)".

When patients in the 'difficult' decision group remained unconvinced by their GP or specialist to take medication, they turned to other sources of information (e.g. friends, family, other health care professionals, pamphlets). In general, these sources of information resulted in a decision *not *to take OP medication. In contrast to participants in the 'easy' decision group who focused on the benefits of the medication, participants in the 'difficult' decision group then engaged in risk-benefit analyses that focused on concerns about potential and actual side effects (See Table 4). For example, one 72-year old female participant was very cautious because she had been told that bisphosphonates cause throat cancer and she concluded that "the fact that they want gallons and gallons of water makes me think that it is doing harm to my throat" (#23). Another participant considered herself extremely well informed and fully understood the severity of her OP but she had done extensive research on the side effects of bisphosphonates and was "not prepared to accept them" (#24-female age 77). Interestingly, this participant described her GP as "an excellent GP" who gave "advice that she thought was best for [her]" but it was her decision to "reject that advice". Yet another participant was very anxious about taking OP medication because she had heard that it could "disintegrate the jaw" and she wanted to make sure she wasn't sacrificing her jaw for her bone health: "I want to balance everything so I'm not dragging down some other part of my health...We are very long lived in my family and I could need my jaw another thirty years" (#3-female age 67).

**Table 4:** Examples of risk/benefit analyses related to the decision to take OP medication

"Easy" Decision		"Difficult" Decision	
Taking OP medication at time of interview	Not taking OP medication at time of interview	Taking OP medication at time of interview	Not taking OP medication at time of interview

*Regarding her decision to take a bisphosphonate*, "It might make my bones stronger,...or I might gain back some bone density" (#18)	*Participant understood she was prescribed a bisphosphonate *"to prevent further bone loss" (#25)	"One friend of mine was asked to take [a bisphosphonate] and she had a major allergic reaction to it and it caused some problems with her blood cells...A neighbour mentioned she'd been on [a bisphosphonate] for almost a year...and she was having some cramping problems in her toe" (#10)	"I did a bit of research and elected not to go on [bisphosphonates] because I didn't feel I wanted to accept the side effects. And I was certainly right...If you look at the side effects of [the bisphosphonate], which has now been discontinued because of jaw necrosis...they lose bone in the jaw...common sense tells one that if you have to swallow a pill and cannot lie down or bend for half an hour because if it lodges in the esophagus, it creates a great deal of damage there...and does the damage where you do not feel it until it's too late" (#24)

The non-permanent nature of the decision to take OP medication was also evident in participants in the "difficult decision" group. One participant (#15-female age 84) who was taking a bisphosphonate at the time of the interview was not certain if, and when, she would refill her prescription. A month's supply of her OP medication remained but she had been cancelling her follow-up appointments with the specialist until she had had time to follow other recommendations such as exercise and calcium intake. Of the 5 patients in this group who were *not *taking OP medication at the time of the interview (1 male age 65, 4 females age 65-77), 3 had a history of bisphosphonate use. One 65-year old male participant was a GP who reported experiencing severe side effects from the bisphosphonate so he had stopped taking it. He did not consider his low bone mass as a serious health concern but was considering switching to the yearly injections of bisphosphonate (#12). One patient with OP had stopped taking two different bisphosphonates after initiating them at separate times, finding them both to be very inconvenient: "with all of them, you can't lie down after for half an hour or something and you have to drink a whole pile of water. In the morning, we take our little grandson to the babysitter. We're up at 6:25 am and I don't have time for drinking gallons of water" (#23-female age 72). Despite encouragement from her GP who was an old friend from university, she didn't think of her OP as a serious health concern. Interestingly, she had been misleading her GP to believe that a specialist at the study hospital was looking after her bone health: "So, I think he [the GP] thinks [the hospital] is looking after it". This patient acknowledged that she had not corrected her GP about this perception.

Similarly, another participant had been urged repeatedly by her GP to take OP medication after she had been first diagnosed with OP 20 years previously; she had tried both the weekly and the yearly bisphosphonate but had decided to stop taking both, comparing her decision to "Russian roulette". She had recently attended two consultations with a specialist but still refused to re-start the medication. However, she told us if she had another fracture, she might reconsider taking OP medication (#24-female age 77).

Two other participants who were not taking OP medication at the time of the interview remained undecided (2 females age 65-67) and had not yet filled their prescriptions for a bisphosphonate. Participant #3 (age 67) had experienced two fractures and her low bone mass was a serious health concern for her; she worried that she might break a hip. But she had allergies and was concerned about what she "put into [her] body". She told us that she would make a decision to take OP medication after consulting with her dentist (she was concerned about losing bone in her jaw). The other participant (#22-age 65) told us: "At this point, I haven't made the decision to take it. That's the decision that would be made. It's not a decision *not *to take it, it would be a decision to take." This participant told us that there was a possibility that she might change her mind - "I might think differently at 70".

## Discussion

This study highlights that for over half of fracture patients, the decision to take OP medication was an easy one. Most of these patients were taking a bisphosphonate. However, the remaining patients reported that the decision required considerable contemplation. The role of specialists and GPs was influential both positively and negatively in the decision to take OP medication. Regardless of whether the decision was a difficult one or not, many participants (n = 11 including 2 who were undecided) indicated that this decision was not permanent and that they might be persuaded to start or stop taking medication depending on a number of circumstances.

The role of health care providers' influence in medication decision-making from a patient perspective is relatively unexplored in OP. In other conditions, Dowell and Hudson [30] characterized primary care patients with a range of medication prescriptions as "passive users", "active users", or "rejectors". They described "passive acceptors", most of whom were elderly, as patients who 'blindly' followed their physician's advice. But their study examined medication-taking *behaviour *and did not elaborate on the decision-making of patients. In mental health, Nolan and Badger [31] illustrated the power of the initial physician consultation to influence patients' beliefs about the appropriateness of antidepressant medication. Patients in Nolan and Badger's study emphasized that regular follow-up, monitoring, and information by their physician to review how treatment was progressing was viewed as symbolizing interest in their well-being.

Many of our participants complained about the side effects (potential and actual) of their bisphosphonate and described how these concerns influenced their decision. This finding is consistent with that of a published synthesis of qualitative studies on medicine taking [32]. However, Brookhart et al. [33] found that the majority of patients who stopped and re-started OP medication, re-started the *same *OP medication suggesting that actual side effects experienced may not be the most important issue for all patients. The results of our study suggest that perceived side effects *are *critical to decision-making, but that these decisions are not static. Ongoing risk/benefit analyses may result in side effects being perceived as acceptable or unacceptable risks. In contrast to Weiss and colleagues [13], who found that effectiveness and time on the market of a medication predicted the decision by patients with, or at risk for, OP, to consider OP medications, the majority of participants in our study did not discuss effectiveness or time on the market as important in their decision to take OP medication.

The non-static nature of the decision in over 50% (11/21) of our patients is partly reassuring, while also giving cause for concern. On the one hand, many participants were receptive to information and, if their concerns were addressed, often indicated they would take medication. Of concern is that patients need to be on antiresorptive medication for at least 6 months for decreased fracture risk to be realized [34]. By 24 months of antiresorptive medication, risk of a hip fracture can be reduced by up to 45% [35]. Our findings indicating the non-static nature of medication decisions suggest that the effectiveness of OP medications may be compromised in many cases. These findings echo those of other studies that find up to 58% of patients who have an indication do not initiate antiresorptive medication after post-fracture OP screening [8], 74% stop taking their medication within 12 months of initiating it [10], and many individuals take OP medication sporadically [36]. However, of patients with and without a fracture who stop OP medication, an estimated 30% re-start within 6 months and 50% re-start within 2 years suggesting that many patients return to OP medication after periods of non-use [33]. We believe that greater emphasis needs to be paid to educating individuals about taking medication over the long term in order to realize benefits. In addition, our findings suggest the need for health care providers to revisit the decision with patients to determine if 'willingness' has changed, and to intervene if this is the case.

There are several implications of our study. Health care providers should be aware of their potential influence on patients' medication-taking decisions and recognize that some patients will decide to take their medications based solely on having a positive relationship with their physician; others will need to be convinced to take medication and discussions should address potential side effects of the OP medications. Post-fracture, patients may also be confused about other aspects of OP medication such as duration of treatment [37] and health care providers should be prepared to address these misconceptions as well. Consistent with recommendations by McCormack & Loewen [11], we propose that patients' values and preferences should be incorporated into OP clinical practice guidelines in order to involve patients in informed decision making. It is also possible that Cranney and colleagues' [38] OP decision aid, that includes information about benefits and risks of OP therapeutic options, could be applied to patients who have had a fracture and are at high risk for future fracture.

Our findings may also inform adherence indices such as the mean possession ratio (MPR). Individuals who do not refill their medications regularly may be constantly engaging in the decision-making process to the extent of reducing their MPR. Consequently, the labels "adherent" vs. "non-adherent" to OP medication may not be a useful indication of medication-taking behaviour because the decision to take a bisphosphonate is not necessarily a permanent one. Health care providers should persist with educating patients who have not initiated medication post-fracture or who have stopped taking it as this decision to take medication is likely to change in future. Finally, patients might benefit from interventions when they experience side effects; information on side effects may support patients' confidence in treatment and offset early discontinuation of OP medication [39].

We acknowledge several limitations to our study. We relied on self-report and did not have other corroborating information such as the health care provider perspective. However, we were not so much interested in the accuracy of information but rather the decisions made as a result of perceptions of OP and information from other sources. We did not follow patients' decision making over time so our findings about the dynamic process of the decision to take OP medication were based on recall and predictions about future behaviour. OP treatment includes other modalities such as calcium, vitamin D, and falls prevention; however, we focused on the decision to take OP medication only. While we did not have access to patient's pharmacy records, our study is strengthened by the fact that the majority of the interviews were conducted in the home where the interviewer was able to verify the bisphosphonate label on patients' medicine bottles. To further strengthen our study, we have provided quotations in participants' own words to demonstrate our findings.

## Conclusions

In conclusion, almost half of our patients with a fragility fracture who were deemed at high risk for another fracture reported finding the decision to take OP medication a 'difficult' one. Regardless of whether the decision was an 'easy' or 'difficult' one, many participants indicated that this decision was not permanent. Health care providers should understand the different manners in which patients make decisions to take prescribed OP medication and their role in these decisions and they must ensure they discuss the potential risks and benefits of OP medication with their patients. Health care providers should also monitor patients' acceptance of their decisions over time as these decisions are likely to change.

## List of abbreviations used

OP: osteoporosis

## Competing interests

The authors declare that they have no competing interests.

## Authors' contributions

JS conceived of and designed the study, participated in the analysis and interpretation of the data, and drafted the manuscript. MG and GH participated in the analysis and interpretation of the data and revised the manuscript for important intellectual content. LF conducted the interviews, participated in the analysis and interpretation of the data, and revised the manuscript for important intellectual content. DB, EB, and VE participated in the interpretation of the data and revised the manuscript for important intellectual content. All authors read and approved the final manuscript.

## Pre-publication history

The pre-publication history for this paper can be accessed here:

http://www.biomedcentral.com/1471-2474/12/92/prepub
